# An innovative method to acquire the location of point A for cervical cancer treatment by HDR brachytherapy

**DOI:** 10.1120/jacmp.v17i6.6355

**Published:** 2016-11-08

**Authors:** Liyun Chang, Sheng‐Yow Ho, Shyh‐An Yeh, Tsair‐Fwu Lee, Pang‐Yu Chen

**Affiliations:** ^1^ Department of Medical Imaging and Radiological Sciences I‐Shou University Kaohsiung Taiwan; ^2^ Department of Nursing Chang Jung Christian University Tainan Taiwan; ^3^ Department of Radiation Oncology Chi Mei Medical Center Liouying Tainan Taiwan; ^4^ Department of Radiation Oncology E‐Da Hospital Kaohsiung Taiwan; ^5^ Medical Physics and Informatics Laboratory Department of Electronics Engineering National Kaohsiung University of Applied Sciences Kaohsiung Taiwan; ^6^ Department of Radiation Oncology Sinlau Christian Hospital Tainan Taiwan

**Keywords:** brachytherapy, cervical cancer, point A, location error

## Abstract

Brachytherapy of local cervical cancer is generally accomplished through film‐based treatment planning with the prescription directed to point A, which is invisible on images and is located at a high‐dose gradient area. Through a standard reconstruction method by digitizing film points, the location error for point A would be 3 mm with a condition of 30° curvature tandem, which is 10° away from the gantry rotation axis of a simulator, and has an 8.7 cm interval between the flange and the isocenter. To reduce the location error of the reconstructed point A, this paper proposes a method and demonstrates its accuracy. The Cartesian coordinates of point A were derived by acquiring the locations of the cervical os (tandem flange) and a dummy seed located in the tandem above the flange. To verify this analytical method, ball marks in a commercial “Isocentric Beam Checker” were selected to simulate the two points A, the os, and the dummies. The Checker was placed on the simulator couch with its center ball coincident with the simulator isocenter and its rotation axis perpendicular to the gantry rotation axis. With different combinations of the Checker and couch rotation angles, the orthogonal films were shot and all coordinates of the selected points were reconstructed through the treatment planning system and compared with that calculated through the analytical method. The position uncertainty and the deviation prediction of point A were also evaluated. With a good choice of the reference dummy point, the position deviations of point A obtained through this analytical method were found to be generally within 1 mm, with the standard uncertainty less than 0.5 mm. In summary, this new method is a practical and accurate tool for clinical usage to acquire the accurate location of point A for the treatment of cervical cancer patient.

PACS number(s): 87.55.km

## I. INTRODUCTION

High‐dose‐rate (HDR) brachytherapy (BT) employed in the treatment of cervical carcinoma has been established for several decades.^1^, ^2^, ^3^, ^4^, ^5^ For curative treatments of all stages, BT plays an essential role in giving patients needed boost doses.^(3,4,6–8)^ The curative potential of radiation therapy for cervical cancer has also been demonstrated to be greatly enhanced by the treatment of intracavitary BT.^9^, ^10^, ^11^, ^12^ By delivering a substantially high dose to the tumor in the central pelvis, while sparing the nearby organs at risk due to the rapid dose falloff,^(13)^ BT leads to an improvement in the patient survival rate with a decrease in the recurrence rate.^(9,14–16)^ Throughout the abundant clinical experience accumulated by radiation oncologists, delivery of a certain dose to point A is still a commonly used prescription for cervical cancer BT.^17^, ^18^, ^19^ Traditionally, the treatment planning is performed through the reconstructed dummy seed positions within the applicators and the prescribed point doses from two orthogonal film images,^(5)^ in which the isodose lines passing through point A form a pear shape encompassing the intended boost treatment volume.^(20)^

Historically, several definitions have been used to define the location of point A in terms of its location along the direction of the tandem (intrauterine applicators). In the earliest Manchester system,^(21)^ point A was defined as “2 cm lateral to the central canal of the uterus, and 2 cm up from the mucous membrane of the lateral fornix in the axis of the uterus”. The definition of point A in 1953 was modified as a point 2 cm superior to the external cervical os and 2 cm lateral to the cervical canal.^(22)^ This modified definition is still referenced in standard medical physics textbooks.^(23)^ Lately, however, the earliest definition of point A was readopted with some adjustments by the American Brachytherapy Society (ABS)^5^, ^24^, ^25^ and European Society for Therapeutic Radiation Oncology (ESTRO).^8^, ^26^

The AAPM TG 56^(27)^ has recommended that the physicist should maintain consistency between past and current practice with respect to the point A dose and critical organ doses. One project of EQUAL‐ESTRO reported that “a 0.5 mm deviation in distance relative to a treatment distance of 20 mm in brachytherapy means a 5% variation in dose delivery”.^(28)^ Moreover, another ESTRO study in HDR BT discussed the high‐dose gradient around point A, stating that “the dose along an axis perpendicular to the intrauterine source at the level of point A decreases from approximately 200% to 100% of the dose to point A when going from 10 to 20 mm from the source, whereas the dose decreases from 100% to approximately 60% from 20 to 30 mm”.^(26)^ Furthermore, Zhang et al.^(29)^ reported that a 9 mm shift in point A can cause a 14% dose rate difference for low‐dose‐rate brachytherapy. Therefore, to obtain the correct location of point A for each individual treatment is extremely important, since a slight variation of its location can result in significant dose variation.^17^, ^29^, ^30^

2D X‐ray imaging is still widely used to calculate the position of point A,^(31)^ which is generally reconstructed through the point marks predrawn on the orthogonal radiographs.^32^, ^33^, ^34^ However, point A is defined in relation to important anatomic structures, but cannot be visualized on a radiograph.^(20)^ Its location cannot be exactly determined through a radiograph also, mainly due to its unknown magnification on film. According to our previous study,^(35)^ if the tandem curvature angle is 30° with a 10° rotation away from the gantry rotation axis in the AP view, and the flange is 8.7 cm away from the isocenter, then the location error of point A and the associated dose error would be 3.0 mm and higher than 8%, respectively. This error could be even higher, since point A is located at a high‐dose gradient area and 8% is the minimum predicted value. In this study, an analytical method to calculate the coordinates of point A is proposed through the use of the reconstructed position of the tandem flange and one reference point on the tandem. The location of point A can be more accurately acquired using this method and the related dose error thereby be substantially reduced.

## II. MATERIALS AND METHODS

In our clinic, the definition of the two points A is based on the modified Manchester system, represented as $A_{1}$ and $A_{2}$, the left and right point A on the anterior–posterior (AP) film image (heads‐up), respectively, which are located 2 cm superior to the external cervical os and 2 cm right and left lateral to the patient's cervical canal, respectively. In a standard orthogonal film reconstruction, point A would be delineated starting from the radiopaque flange of the tandem that should be adjacent to the cervical os. It is generally reconstructed in the treatment planning system after carefully digitizing the point marks that were previously drawn on the orthogonal radiographs into the system. We will refer to this procedure as the “standard” method. In this work, we propose an alternative, analytical method, as described below.

**Figure 1:**
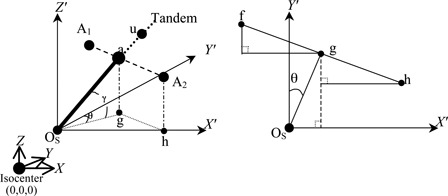
Relative positions of tandem, $O_{S}$, and point A. The $O_{S}$ represents the position of the cervical os with the coordinates ($x_{\text{os}},y_{\text{os}},z_{\text{os}}$); $\bar{O_{S}u}$ is aligned with the tandem (the intrauterine applicator) and is 2 cm superior from the $O_{S}$ to the “a” point (the center of $A_{1}$ and $A_{2}$); θ is the angle between the projection of the tandem on the $x^{\prime }$‐ to $y^{\prime }$‐plane and the $y^{\prime }$‐axis; γ is the angle of between $\bar{O_{S}u}$ and the $x^{\prime }$‐ to $y^{\prime }$‐plane; g, f, and h are the projections of the points a, $A_{1}$, and $A_{2}$ on the $x^{\prime }$‐ to $y^{\prime }$‐plane, respectively.

Preparing the BT treatment for cervical cancer, the patient is placed in a supine position on a movable homemade couch with feet toward the gantry of our Toshiba DC50N simulator (Tokyo, Japan), and then the orthogonal X‐ray images are taken for film reconstruction. To calculate point A, a Cartesian coordinate is defined with the origin at the simulator isocenter, the z‐axis paralleling the gravity but in the opposite direction, the y‐axis paralleling the gantry rotation axis but directed away from the gantry, and the x‐axis pointing towards the patient's left. Another three axes, $x^{\prime },y^{\prime }$, and $z^{\prime }$, starting from a point $O_{s}$ with the coordinates ($x_{\text{os}},y_{\text{os}},z_{\text{os}}$) are defined to have the same directions as the x‐, y‐, and z‐axis, respectively (Fig. 1). The point $O_{S}$ is coincident with the location of the flange (Fig. 1), which is also the assumed position of the cervical os.

During BT, the patient's back is assumed to be lying flat on the couch, so that the line connecting the two points A could be taken as parallel to the $x^{\prime }$‐ to $y^{\prime }$‐plane. The angle between the tandem and the $x^{\prime }$‐ to $y^{\prime }$‐plane is defined as γ degrees (usually this is the curvature angle of the applicator, if the lower part of the applicator paralleled to the $x^{\prime }$‐ to $y^{\prime }$‐plane), where the projection of the tandem on the $x^{\prime }$‐ to $y^{\prime }$‐plane is θ degrees away from the $x^{\prime }$‐axis (Fig. 1). To calculate the location of point A requires the location of $O_{s}$ and a reference point u, which can be a dummy seed with coordinates ($x_{u},y_{u},z_{u}$) located at the tandem above the flange. The coordinates of these two points can be obtained by digitizing their images shown on the orthogonal films and executing the reconstruction using the computer planning system or through manual calculation.^(35)^ According to Fig. 1, the θ, γ and the coordinates at the z‐axis of $A_{1}$ and $A_{2}$ points are given by:

$$\theta ={\text{tan}}^{-1}\left(\frac{x_{u}-x_{os}}{y_{u}-y_{os}}\right)$$

$$\gamma ={\text{sin}}^{-1}\left(\frac{z_{u}-z_{os}}{\sqrt{{\left(x_{u}-x_{os}\right)}^{2}+{\left(y_{u}-y_{os}\right)}^{2}+{\left(z_{u}-z_{os}\right)}^{2}}}\right)$$

$$z_{A1}=z_{A2}=\bar{O_{S}a}\cdot \text{sin}\gamma +z_{os}=2(cm)\cdot \text{sin}\lambda +z_{os}$$

Through Fig. 1(b) with $\bar{O_{S}a}=2\;\text{cm}$, the coordinates at x‐axis and y‐axis of $A_{1}$ and $A_{2}$ points could be written as:

$$x_{A1}=2(cm)\cdot \text{cos}\gamma \cdot \text{sin}\theta -2(cm)\cdot \text{cos}\theta +x_{os}$$

$$x_{A2}=2\cdot \text{cos}\gamma \cdot \text{sin}\theta +2\cdot \text{cos}\theta +x_{os}$$

$$y_{A1}=2\cdot \text{cos}\gamma \cdot \text{cos}\theta +2\cdot \text{sin}\theta +y_{os}$$

$$y_{A2}=2\cdot \text{cos}\gamma \cdot \text{cos}\theta -2\cdot \text{sin}\theta +y_{os}$$

**Figure 2:**
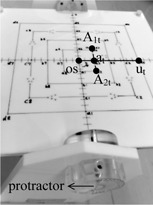
The “Isocentric Beam Checker” device and the points os, $A_{1t}$, at, $A_{2t}$, and $u_{t}$ marked on it.

Then the coordinates of the $A_{1}$ and $A_{2}$ points can be accurately calculated and input into the planning system for the dose calculation. The “Isocentric Beam Checker” device was used to verify the calculations and processes above. As shown in Fig. 2, on top of the Checker there are four balls in each of eight directions (viewed from the center) and one ball located at the center (marked as “$O_{s}$”). All balls have a diameter of approximately 1.5 mm and are embedded on the 2D surface of the Checker. The ball points on one side of the Checker with the smallest carved square (5cm×5cm) were marked as $A_{1t},a_{t}$, and $A_{2t}$ (Fig. 2). Points os, $a_{t}$, and $u_{t}$ were located on the same line in order to mimic the line of a tandem, where $a_{t}$ and $u_{t}$ were 2.5 cm and 10 cm away from the point os, respectively. $A_{1t}$ and $A_{2t}$ were the two tested points A and 2.5 cm away from the simulated tandem. [Link], [Link] were used for calculations of the verification, but “2 (cm)” was replaced by “2.5 (cm)” in [Link], [Link]. The coordinates of $A_{1t}$ and $A_{2t}$ were defined as ($x_{A1t},y_{A1t},z_{A1t}$) and ($x_{A2t},y_{A2t},z_{A2t}$), respectively. Two printed protractor transparencies were adhered on both sides of the rotation bar of the Checker to indicate its rotation angle (Fig. 2).

The Checker was placed horizontally on the simulator couch with its center ball coincident with the simulator isocenter, and the line with the marks “os” and “$u_{t}$” on it was also coincident with the axis of gantry rotation. The distance between point os and simulator isocenter was represented as ρ_os_, and ideally it is zero here. Then we adjusted the γ angle by rotating the Checker to be 20°, 30°, and 40° according to the index of the tabbed protractor relative to the indication of the laser projection. With the γ angle fixed, the couch angle θ was set to be 10°, 20°, and 30°. For those setups, a total of 18 films (nine AP films and nine lateral films) for reconstruction were shot and developed. All the points on films, os, $A_{1t},a_{t},A_{2t}$, and $u_{t}$, were digitized and reconstructed through the Abacus treatment planning system (MDS Nordion, Rostok, Germany, version 3.1) and all the information was used for further analysis.

After reconstruction, the distance deviation ($\Delta d_{0}$) of the points $A_{1t}$ and $A_{2t}$ between the reconstructed positions from the planning system and the theoretically calculated positions through above equations can be written as:

$$\Delta d_{0}=\sqrt{\Delta x^{2}+\Delta y^{2}+\Delta z^{2}}$$

where the reconstructed coordinates of $A_{1t}$ and $A_{2t}$ are ($x_{A1t}^{\prime },y_{A1t}^{\prime },z_{A1t}^{\prime }$) and ($x_{A2t}^{\prime },y_{A2t}^{\prime },z_{A2t}^{\prime }$), respectively; the theoretically calculated coordinates of $A_{1t}$ and $A_{2t}$ are ($x_{A1t},y_{A1t},z_{A1t}$) and ($x_{A2t},y_{A2t},z_{A2t}$), respectively; for point $A_{1t},\Delta x=x_{A1t}^{\prime }‐x_{A1t},\Delta y=y_{A1t}^{\prime }‐y_{A1t}$, and $\Delta z=z_{A1t}^{\prime }‐z_{A1t}$; for point $A_{2t},\Delta x=x_{A2t}^{\prime }‐x_{A2t},\Delta y=y_{A2t}^{\prime }‐y_{A2t}$, and $\Delta z=z_{A2t}^{\prime }‐z_{A2t}$.

Using the report, recommendations from the gynecological (GYN) GEC ESTRO Working Group (II) of 2006:^(26)^ “The dose along an axis perpendicular to the intrauterine source at the level of point A decreases from approximately 200% to 100% of the dose to point A when going from 10 to 20 mm from the source, whereas dose decreases from 100% to approximately 60% from 20 to 30 mm.” If we simply took the average of dose variation for the two directions, away or toward the source, we could conclude that “the dose variation along the axis perpendicular to the intrauterine source at the level of point A can be approximately estimated as, with respect to the dose at point A, 10% increase per mm or 4% decrease per mm toward or away from the source, respectively.”^(35)^ Taking the tandem as approximately parallel to the y‐axis, the dose gradient along the line that passes through the point A on the x‐ to z‐plane would be more important and could be taken as essentially the same dose gradient along the axis described in the previous sentence. The distance deviation on the x‐ to z‐plane, represented by $\Delta_{\text{dxz}0}$, for point $A_{1t}$ and $A_{2t}$ is given by:

$$\Delta d_{xz0}=\sqrt{\Delta x^{2}+\Delta z^{2}}$$

To make an effective choice of the reference point, the point $u_{t}$ was tested by two different locations: one at the same location of point $a_{t}$, where the distance between $u_{t}$ and os is 2.5 cm, and the other at a position 10 cm away from the os (Fig. 2).

**Figure 3:**
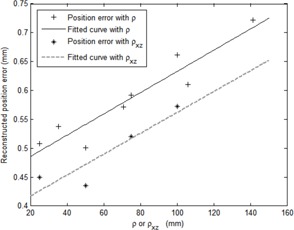
Reconstructed position error and the fitted curve in space or on the x‐ to z‐plane for a point located ρ mm away from the isocenter or the point $\rho_{\text{xz}}\;\text{mm}$ away from the isocenter on the x‐ to z‐plane, respectively.

In clinical practice, the patient os is generally not coincident with the simulator isocenter, so the position error of the os point on plate, if away from the isocenter, was estimated using the results of our previous work.^(34)^ According to Figure 4 in Chang et al.,^(34)^ for a point with a distance of ρ mm away from the isocenter (its projection on x‐ to z‐plane represented as $\rho_{\text{xz}}$), the position error in space and on the x‐ to z‐plane are illustrated in Fig. 3, which is based on the quality assurance results of ±0.1cm for the source‐to‐film distance (SFD) and ±0.1° for the angle indicators of the gantry and collimator.

The position error of os in space ($\Delta_{\text{dos}}$) and that on the x‐ to z‐plane ($\Delta_{\text{dxz},\text{os}}$) can be fitted with a single‐order polynomial in ρ and $\rho_{\text{xz}}$, respectively. Each of them can be given by:

$$\Delta d_{os}=k_{1}\times \rho +k_{2}$$

and

$$\Delta d_{xz,os}=k_{3}\times \rho_{xz}+k_{4}$$

where $k_{1},k_{2},k_{3}$, and $k_{4}$ are fitting parameters calculated using the MATLAB software (MathWorks, Natick, MA) with the values of 0.0018, 0.4492, 0.0018, and 0.3814, respectively; ρ and $\rho_{\text{xz}}$ are in units of mm. Then the combined position error for a reconstructed ball point can be written as:

$$\Delta d=\sqrt{\Delta d_{0}^{2}+\Delta d_{os}^{2}}$$

and the combined error on the x‐ to z‐plane is given by:

$$\Delta d_{xz}=\sqrt{\Delta d_{xz0}{\;}^{2}+\Delta d_{xz,os}{\;}^{2}}$$

where $\Delta_{d0}$ and $\Delta d_{\text{xz}0}$ are defined in Eqs. (8) and (9).

### A. Uncertainty and deviation prediction for the location of point A

To perform the uncertainty analysis, [Link], [Link] were rewritten by substituting the θ and γ with Eqs. (1) and (2), respectively, in them:

$$z_{A1}=z_{A2}=f_{z}(x_{os},y_{os},z_{os},x_{u},y_{u},z_{u})+z_{os}$$

$$x_{A1}=f_{X\_A1}(x_{os},y_{os},z_{os},x_{u},y_{u},z_{u})+x_{os}$$

$$x_{A2}=f_{X\_A2}(x_{os},y_{os},z_{os},x_{u},y_{u},z_{u})+x_{os}$$

$$y_{A1}=f_{Y\_A1}(x_{os},y_{os},z_{os},x_{u},y_{u},z_{u})+y_{os}$$

$$y_{A1}=f_{Y\_A2}(x_{os},y_{os},z_{os},x_{u},y_{u},z_{u})+y_{os}$$

where the *f* is a function representing the distance between points A and $O_{s}$ on each axis; and the subscript of *f* indicates the axis and one of the two points A. The uncertainty of the f, Δf, in [Link], [Link], can be calculated through a numerical method listed on page 19 of the report “GUM: Guide to the expression of uncertainty in measurement”,^(36)^ which is given by:

$$y_{A1}=f_{Y\_A2}(x_{os},y_{os},z_{os},x_{u},y_{u},z_{u})+y_{os}$$

where $x_{1},x_{2},x_{3},x_{4},x_{5}$, and $x_{6}$ represent $x_{\text{os}},y_{\text{os}},z_{\text{os}},x_{u},y_{u}$, and $z_{u}$, respectively; Δ$x_{i}$ is the standard uncertainty of the variable $x_{i}$; for instance, the standard uncertainty of the variable $z_{\text{os}}$ is $\Delta z_{\text{os}}$. In addition $\Delta f_{Z},{\Delta f}_{X\_A1},{\Delta f}_{Y\_A1},{\Delta f}_{X\_A2}$, and ${\Delta f}_{X\_A2}$ are the standard deviations calculated through Eq. (19) with the f equal to $f_{Z},{\Delta f}_{X\_A1},{\Delta f}_{Y\_A1},{\Delta f}_{X\_A2}$, and ${\Delta f}_{Y\_A2}$, respectively. When performing the reconstruction work, based on our previous report,^(33)^ the standard deviation of a reconstructed point in our facility deviating from its theoretical position is 0.26, 0.21, and 0.26 mm in the x‐, y‐, and z‐axis, respectively. Therefore, to predict the deviation for $A_{1}$ and $A_{2}$ in our system, all of the $\Delta x_{i}$ values were assigned the value of 0.26 mm, except for $\Delta y_{\text{os}}$ and $\Delta y_{u}$, which were assigned the value of 0.21 mm.

The standard uncertainties of $x_{\text{os}},y_{\text{os}}$, and $z_{\text{os}},\Delta x_{\text{os}},\Delta y_{\text{os}}$, and $\Delta z_{\text{os}}$, were also assigned the values of 0.26, 0.21, and 0.26, respectively. According to [Link], [Link], after combining all the uncertainties in each axis, the standard uncertainties of $A_{1}$ and $A_{2}$ in space were given by:

$$\Delta A_{1}=\sqrt{\left(\Delta f_{X\_A1}{\;}^{2}+\Delta x_{os}{\;}^{2})+(\Delta f_{Y\_A1}{\;}^{2}+\Delta y_{os}{\;}^{2})+(\Delta f_{Z}^{2}+\Delta z_{os}{\;}^{2}\right)}$$

and

$$\Delta A_{2}=\sqrt{\left(\Delta f_{X\_A2}{\;}^{2}+\Delta x_{os}{\;}^{2})+(\Delta f_{Y\_A2}{\;}^{2}+\Delta y_{os}{\;}^{2})+(\Delta f_{Z}^{2}+\Delta z_{os}{\;}^{2}\right)}$$

Similarly, the combined uncertainties of points $A_{1}$ and $A_{2}$ on the x‐ to z‐plane can be given by:

$$\Delta A_{1XZ}=\sqrt{\left(\Delta f_{X\_A1}{\;}^{2}+\Delta x_{os}{\;}^{2})+(\Delta f_{Z}^{2}+\Delta z_{os}{\;}^{2}\right)}$$

and

$$\Delta A_{2XZ}=\sqrt{\left(\Delta f_{X\_A2}{\;}^{2}+\Delta x_{os}{\;}^{2})+(\Delta f_{Z}^{2}+\Delta z_{os}{\;}^{2}\right)}$$

[Link], [Link] were also used for the uncertainty prediction of point $A_{1t}$ and point $A_{2t}$ in the verification test.

## III. RESULTS & DISCUSSION

Combining the deviation prediction ([Link], [Link]), Table 1 shows the distance deviations, $\Delta d_{0}$ (Eq. (8)) and $\Delta d_{\text{xz}0}$ (Eq. (9)), the deviations between the theoretical calculation ([Link], [Link] with the weight of 2 replaced by 2.5) and the reconstructed positions of points $A_{1t}$ and $A_{2t}$ with two different reference points for which $u_{t}$ is 2.5 and 10 cm above the os. The averaged $\Delta d_{0}$ and $\Delta d_{\text{xz}0}$ are approximately 0.8 mm and 0.5 mm, respectively, and their highest values are less than 1.3 mm and 0.9 mm, respectively. For $u_{t}=10$, the reference point 10 cm away from the os, the deviations are consistent with our premeasurements.^(34)^ As previously stated in Chang et al.^(33)^ and Chang et al.,^(34)^ the $\Delta d_{0}$ was primarily contributed to by the inaccuracies of the gantry angle, collimator angle, SFD indicators, and the error of magnification and minimization calculation. [Link], [Link] were then shown to be valid through this verification test. The differences between the deviation prediction and the averaged $\Delta d_{0}$ and $\Delta d_{\text{xz}0}$ in each item are less than 0.1 mm.

With different combinations of θ and γ, the position errors ($\bar{\Delta d}$ and $\bar{d_{\text{xz}}}$) of the tested point A, averaged from the combined position error of points $A_{1t}$ and $A_{2t}$ calculated through Eqs. (12) and (13), are listed in Table 2 and Table 3 for $u_{t}=2.5\;\text{cm}$ and $u_{t}=10\;\text{cm}$, respectively. The $O_{s}$ coordinates in Tables 2 and 3 are represented by (h, h, h) in centimeters. For $u_{t}=2.5\;\text{cm}$, when γ=40° with θ≥20° or h=10, the averaged position errors in space ($\bar{\Delta d}$) are generally greater than 1 mm, but all the errors were less than 1.4 mm. Except for h=10, the averaged position error on the x‐ to z‐plane ($\bar{d_{\text{xz}}}$) is less than 0.9 mm. In Table 2, $\bar{\Delta d}$ is clearly less than 1 mm only for h ≤2.5cm and γ≤40°. All $\bar{d_{\text{xz}}}$ values are less than 1.0 mm in Tables 2 and 3.

For $u_{t}=10\;\text{cm}$ in Table 3, all averaged position errors are less than 1.1 mm; $\bar{\Delta d}$ is less than 1 mm, except for γ=40° with θ=30° or h=10. For γ≤30° and $h=0,\bar{d_{\text{xz}}}$ is less than 0.5 mm. For γ≤30° and $h\leq 5,\bar{d_{\text{xz}}}$ is within 0.7 mm. Comparing Tables 2 and 3, $u_{t}=10\;\text{cm}$ is clearly a better choice than $u_{t}=2.5\;\text{cm}$.

For comparison with the standard method published in Chang et al.,^(35)^ Table 4 lists the position deviation of point $A_{t}$ and point A, calculated using the analytical method (Eqs. (12) and (13)) and the standard method,^(35)^ respectively. For h≥5cm, the deviations of the analytical method will be 1~5mm less than that of the standard method. The deviations of the analytical method are also less affected by coordinate variations of the point os.

**Figure 4:**
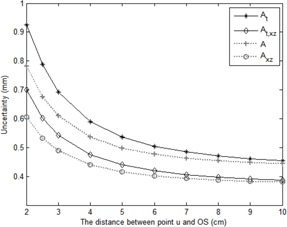
The position uncertainty calculated for point A and tested point Aj in space and on the x‐ to z‐plane with different distances between the os and the reference point by using [Link], [Link].

Using [Link], [Link], with different $\bar{O_{S}u}$ (2~10cm), the interval between the os and the reference point u, Fig. 4 demonstrates the predicted position uncertainty of point A and the tested point $A_{t}$, both in space and on the x‐ to z‐plane, which was averaged from that of the left and right point A. If the chosen u point is 6 cm away from the os, the position uncertainty of point A in space and on the x‐ to z‐plane would be less than 0.5 mm and 0.4 mm, respectively. In that case, the associated dose uncertainty of the prescribed dose to point A would be around 1.6% and 4%, respectively, deduced from the previous statement that toward the tandem there is approximately 4%/mm decrease or 10%/mm increase with respect to the dose at point A, respectively.^(35)^ Theoretically, a larger value of $\bar{O_{S}u}$ will lead to smaller uncertainty; however, as shown by Fig. 4, the uncertainty would not change much if $\bar{O_{S}u}$ is larger than 6 cm.

**Table 1:** The distance deviation ($\Delta d_{0}$ and $\Delta d_{\text{xz}0}$) in millimeters between the theoretical calculation and the reconstructed position of point $A_{1t}$ and $A_{2t}$ in connection with the deviation prediction ([Link], [Link]). The subscripts “$u_{t}=2.5$” and “$u_{t}=10$” represent the distances chosen of $u_{t}\;2.5\;\text{cm}$ and 10 cm away from the os, respectively

θ	*10°*	*20°*	*30°*	*10°*	*20°*	*30°*	*10°*	*20°*	*30°*		*Deviation*
γ	*20°*	*20°*	*20°*	*30°*	*30°*	*30°*	*40°*	*40°*	*40°*	*Average*	*Prediction*
$\Delta_{0,A1t,\text{ut}=2.5}$	1.186	0.319	1.097	0.609	0.494	0.875	0.428	1.210	1.004	0.802	0.788
$\Delta_{0,A2t,\text{ut}=2.5}$	0.441	0.754	0.557	0.907	0.826	0.526	0.408	1.018	0.917	0.706	0.787
$\Delta_{0,A1t,\text{ut}=10}$	0.468	0.518	0.578	0.415	0.477	0.451	0.283	0.577	0.798	0.507	0.455
$\Delta_{0,A2t,\text{ut}=10}$	0.626	0.385	0.134	0.895	0.318	0.569	0.331	0.626	0.657	0.504	0.454
$\Delta_{\text{xz}0,A1t,\text{ut}=2.5}$	0.640	0.300	0.748	0.484	0.456	0.736	0.428	0.757	0.721	0.586	0.681
$\Delta_{\text{xz}0,A2t,\text{ut}=2.5}$	0.441	0.722	0.487	0.889	0.081	0.452	0.328	0.293	0.608	0.478	0.525
$\Delta_{\text{xz}0,A1t,\text{ut}=10}$	0.251	0.437	0.286	0.346	0.241	0.349	0.277	0.556	0.711	0.384	0.395
$\Delta_{\text{xz}0,A2t,\text{ut}=10}$	0.417	0.382	0.051	0.634	0.291	0.568	0.193	0.617	0.651	0.423	0.379

According to Tables 1 to 3, to have less position error of point A, the best choice for point u is the one further from the os, as demonstrated in Fig. 4. Therefore, the physicist had better choose the reference dummy point to be at least 6 cm away from the flange to calculate the coordinate of point A. Compared with the standard method, the analytical method provided substantial improvement to make the position deviation of point A generally less than 1 mm (with good choice of the reference point) and the position uncertainty would be less than 0.5 mm. With appropriate uncertainty prediction, the proposed new technique is a practical and excellent tool for clinical usage to acquire the accurate location of point A and deliver a more accurately prescribed dose to the patient.

**Table 2:** The averaged position errors, $\bar{\Delta d}$ and $\bar{d_{\text{xz}}}$ (in mm), of all test angles with $u_{t}=2.5\;\text{cm}$. The coordinates of point $O_{s}$ (h, h, h) are in cm

*Point O*_*s*_ *Coordinates with u*_*t*_ *=2.5 cm*	θ	*10°*	*20°*	*30°*	*10°*	*20°*	*30°*	*10°*	*20°*	*30°*
	γ	*20°*	*20°*	*20°*	*30°*	*30°*	*30°*	*40°*	*40°*	*40°*
(0, 0, 0)	$\bar{\Delta d}$	0.813	0.537	0.827	0.758	0.660	0.700	0.418	1.114	0.961
(0, 0, 0)	$\bar{\Delta_{\text{xz}}}$	0.541	0.511	0.618	0.686	0.269	0.594	0.378	0.525	0.664
(2.5, 2.5, 2.5)	$\bar{d_{\text{xz}}}$	0.970	0.753	0.982	0.924	0.845	0.878	0.674	1.233	1.096
(2.5, 2.5, 2.5)	$\bar{d_{\text{xz}}}$	0.700	0.678	0.761	0.818	0.520	0.742	0.584	0.689	0.800
(5, 5, 5)	$\bar{\Delta d}$	1.016	0.811	1.027	0.972	0.897	0.928	0.738	1.269	1.137
(5, 5, 5)	$\bar{\Delta d_{\text{xz}}}$	0.743	0.721	0.800	0.854	0.576	0.782	0.634	0.732	0.837
(7.5, 7.5, 7.5)	$\bar{\Delta d}$	1.065	0.872	1.076	1.023	0.953	0.982	0.805	1.309	1.181
(7.5, 7.5, 7.5)	$\Delta d_{\text{xz}}$	0.788	0.768	0.842	0.894	0.633	0.825	0.686	0.777	0.877
(10, 10, 10)	$\bar{\Delta d}$	1.118	0.936	1.128	1.079	1.012	1.039	0.874	1.352	1.229
(10, 10, 10)	$\Delta d_{\text{xz}}$	0.835	0.816	0.887	0.936	0.691	0.871	0.740	0.825	0.920

**Table 3:** The averaged position error, $\bar{\Delta d}$ and $\bar{d_{\text{xz}}}$ (in mm), of all test angles with $u_{t}=10\;\text{cm}$. The coordinates of point $O_{s}$ are in cm

*Point O*_*s*_ *Coordinates with u*_*t*_ *=2.5 cm*	θ	*10°*	*20°*	*30°*	*10°*	*20°*	*30°*	*10°*	*20°*	*30°*
	γ	*20°*	*20°*	*20°*	*30°*	*30°*	*30°*	*40°*	*40°*	*40°*
(0, 0, 0)	$\bar{\Delta d}$	0.547	0.451	0.356	0.655	0.397	0.510	0.307	0.602	0.728
(0, 0, 0)	$\bar{d_{\text{xz}}}$	0.334	0.410	0.168	0.490	0.266	0.459	0.235	0.586	0.681
(2.5, 2.5, 2.5)	$\bar{\Delta d}$	0.761	0.695	0.637	0.842	0.661	0.734	0.611	0.801	0.899
(2.5, 2.5, 2.5)	$\bar{d_{\text{xz}}}$	0.556	0.605	0.476	0.662	0.519	0.639	0.504	0.736	0.814
(5, 5, 5)	$\bar{\Delta d}$	0.818	0.757	0.705	0.894	0.726	0.794	0.681	0.856	0.948
(5, 5, 5)	$\bar{d_{\text{xz}}}$	0.609	0.653	0.536	0.707	0.574	0.685	0.561	0.777	0.850
(7.5, 7.5, 7.5)	$\bar{\Delta d}$	0.879	0.823	0.774	0.950	0.794	0.856	0.753	0.914	1.001
(7.5, 7.5, 7.5)	$\bar{d_{\text{xz}}}$	0.663	0.704	0.597	0.754	0.632	0.734	0.619	0.820	0.890
(10, 10, 10)	$\bar{\Delta d}$	0.942	0.890	0.846	1.009	0.864	0.921	0.826	0.975	1.057
(10, 10, 10)	$\bar{d_{\text{xz}}}$	0.719	0.757	0.659	0.803	0.690	0.785	0.679	0.866	0.932

**Table 4:** Position deviations ($\bar{\Delta d}$ and $\bar{d_{\text{xz}}}$) in mm) of point $A_{t}$ obtained through the analytical method (data from Table 3) compared with that of the point A obtained through the standard method (data from Table 1 in Chang et al.^(35)^)

*Method Point* $O_{s}$ *Coordinate*		*Analytical*	*Standard*	*Analytical*	*Standard*	*Analytical*	*Standard*	*Analytical*	*Standard*
		θ=10°		θ=20,		θ=10°		θ=20°	
		γ=20°		γ=20°		γ=30°		γ=30°	
(0, 0, 0)	$\bar{\Delta d}$	0.55	1.94	0.45	3.61	0.66	1.94	0.40	3.62
(0, 0, 0)	$\bar{d_{\text{xz}}}$	0.33	0.19	0.41	0.20	0.49	0.28	0.27	0.28
(2.5, 2.5, 2.5)	$\bar{\Delta d}$	0.76	2.33	0.70	3.99	0.84	2.37	0.66	4.02
(2.5, 2.5, 2.5)	$\bar{d_{\text{xz}}}$	0.56	0.97	0.61	0.99	0.66	1.14	0.52	1.21
(5, 5, 5)	$\bar{\Delta d}$	0.82	2.90	0.76	4.45	0.89	3.00	0.73	4.57
(5, 5, 5)	$\bar{d_{\text{xz}}}$	0.61	1.75	0.65	1.80	0.71	2.04	0.57	2.18
(7.5, 7.5, 7.5)	$\bar{\Delta d}$	0.88	3.60	0.82	5.00	0.95	3.80	0.79	5.25
(7.5, 7.5, 7.5)	$\bar{d_{\text{xz}}}$	0.66	2.54	0.70	2.60	0.75	2.96	0.63	3.18
(10, 10, 10)	$\bar{\Delta d}$	0.94	4.43	0.89	5.65	1.01	4.72	0.86	6.06
(10, 10, 10)	$\bar{d_{\text{xz}}}$	0.72	3.32	0.76	3.41	0.80	3.89	0.69	4.20

## ACKNOWLEDGMENTS

This work was supported in part by the Ministry of Science and Technology of Taiwan (NSC 102‐2221‐E‐214‐004) and (MOST 104‐2221‐E‐214‐014).

## COPYRIGHT

This work is licensed under a Creative Commons Attribution 3.0 Unported License.
